# An efficient hypochlorous acid fluorescence imaging strategy based on toehold-mediated strand displacement for distinguishing cancer cells from normal cells

**DOI:** 10.1039/d6ra08265c

**Published:** 2026-10-30

**Authors:** Qianqian Duan, Aihua Xu, Ruobing Han, Guochao Xu, Qianqing Wang, Changbei Ma

**Affiliations:** a Department of Clinical Laboratory, Xinxiang Central Hospital 453000 Xinxiang Henan China; b School of Life Sciences, Central South University 410013 Changsha Hunan China macb2012@csu.edu.cn; c Central Laboratory, Xinxiang Central Hospital 453000 Xinxiang Henan China Wangq1111@126.com; d Department of Gynecology Oncology, Xinxiang Central Hospital 453000 Xinxiang Henan China

## Abstract

Cancer is among the most significant diseases endangering global public health. Early detection has been demonstrated to markedly improve prognoses and increase cure rates. Hypochlorous acid (HClO) is a pivotal reactive oxygen species, frequently manifesting elevated levels in cancer cells. Herein, a highly sensitive, highly specific, and efficient HClO detection method was established based on the toehold-mediated strand displacement (TMSD). This method employs a duplex complex (TN) formed from three single-stranded DNAs to carry a fluorescent group (FAM), and introduces a hairpin DNA (HP) with a phosphorothioate (PS)-modified site for specific recognition of HClO. Under the action of HClO, the short fragment bearing FAM group is eventually released from TN, resulting in a significant increase in the system fluorescence. After optimization, the method achieves excellent HClO detection performance, with a detection range of 0.01–7 µM (LOD: 12.9 nM). Analysis of real samples, including tap water, serum and cell lysate, also confirms its good stability and accuracy. Most importantly, the method enables imaging of intracellular HClO, thereby effectively distinguishing cancer cells from normal cells. Overall, this method offers various advantages, including excellent detection performance, fast response times, ease of use, and readily available materials, making it promising for application in early diagnosis of cancer.

## Abstract 2

A toehold-mediated strand-displacement DNA sensor is constructed for sensitive fluorescence imaging of hypochlorous acid, enabling discrimination between cancer and normal cells.

## Graphical abstract

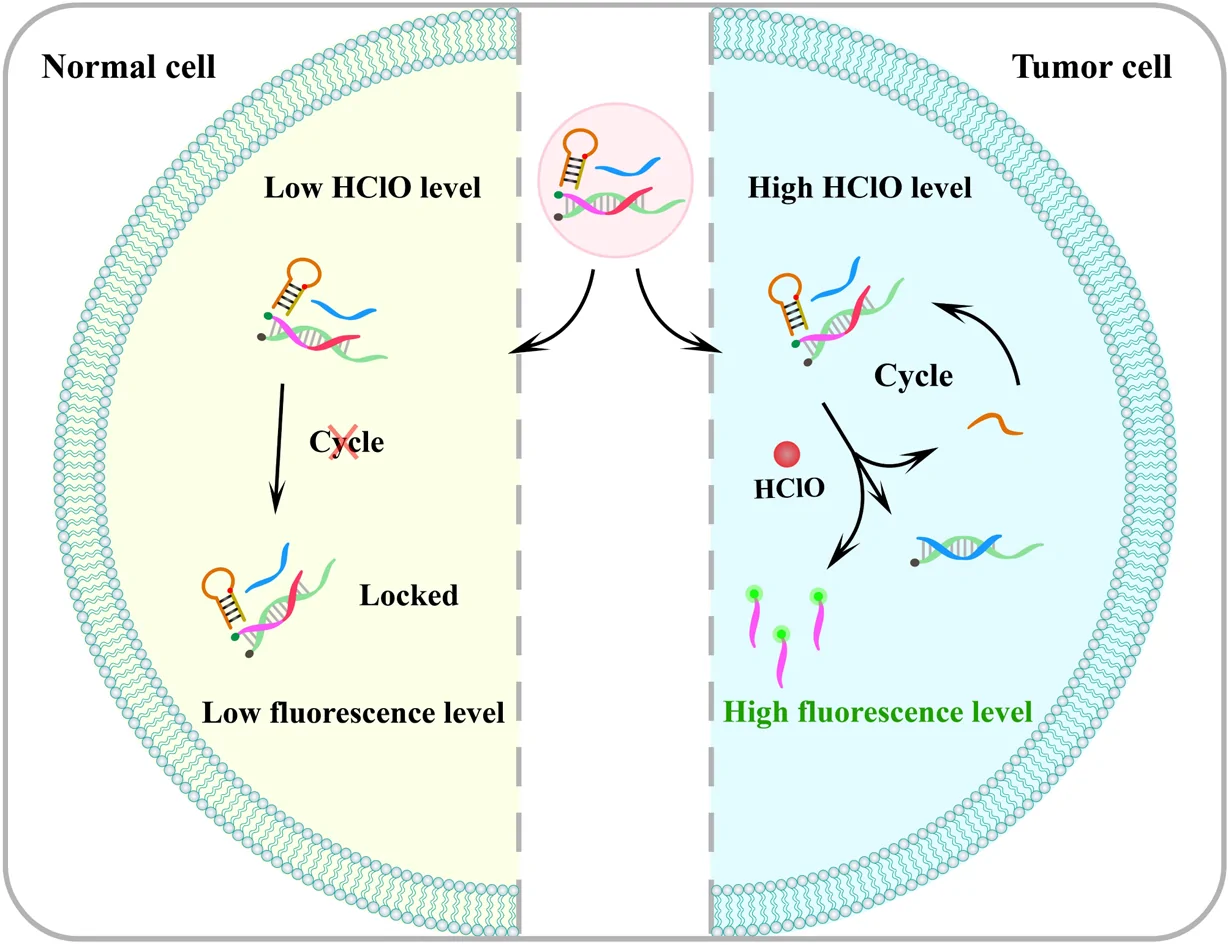

## Introduction

Cancer is a highly lethal disease on a global scale, posing a grave threat to human health. In recent years, a trend towards earlier-onset cancer has been observed, with the global cancer burden among adolescents and young adults continuing to rise. It creates a challenging environment for prevention and control efforts.^1–3^ Many studies have demonstrated that the nebulous nature of early-stage cancer symptoms usually results in delayed diagnoses and treatments, which can ultimately lead to malignant progression. Conversely, an early and precise diagnosis can markedly improve prognosis and increase patient survival rates.^4–6^ However, numerous barriers currently impede the implementation of early cancer screening, including the limited sensitivity and accuracy of existing diagnostic technologies. This creates an urgent need to refine or establish new diagnostic systems.^7,8^ A comparison of normal cells and cancer cells reveals significant biochemical disparities. Among these disparities are a dysregulation of oxidative stress homeostasis, causing aberrantly elevated levels of reactive oxygen species (ROS), and an accumulation of reactive nitrogen species (RNS), such as peroxynitrite. Additionally, there is an increased expression of certain enzymes. These characteristics can serve as key markers for distinguishing cancer cells.^9–13^

Hypochlorous acid (HClO) is a pivotal reactive oxygen species within living organisms, generated *via* myeloperoxidase catalysis. It is a key component in many physiological processes, including immune defense, oxidative stress regulation, and cellular signaling.^14^ Fluctuations in HClO levels directly reflect the state of cellular oxidative stress. An imbalance in this homeostasis can lead to oxidative damage to molecules such as proteins, lipids, and nucleic acids, triggering various pathological processes.^15^ Numerous research has shown that abnormally elevated HClO levels exhibit a strong correlation with many pathological conditions, including inflammation, liver damage, atherosclerosis, and cancer.^16–21^ In the context of cancer research, HClO levels in cancer cells have been observed to be considerably elevated in comparison to those in normal cells. This makes it a critical biomarker, with the potential to differentiate between cancer cells and normal cells. Abnormally accumulated HClO can intensify oxidative stress, promote cancer cell proliferation and invasion, and drive malignant tumor progression.^11,22–25^ Consequently, the precise detection of intracellular HClO is significant for the early screening of cancer cells and the reduction of malignant outcomes. The prevailing HClO detection methods are predicated on fluorescent probe technology, with a range of probes, including near-infrared, ratio-based, and dual-functional types, having been developed. However, these methods still exhibit many limitations, including insufficient specificity, low sensitivity, slow response times, poor stability *in vivo* imaging, and low resolution.^26–30^ Thus, the establishment of novel and effective detection methods is imperative.

At present, nucleic acid probe technology has become a core method in biochemical analysis and bioimaging due to its excellent programmability and biocompatibility. It has been widely applied in fields such as disease diagnosis and treatment.^31,32^ Various strategies have been devised for signal amplification under enzymatic-free and isothermal conditions, including toehold-mediated strand displacement (TMSD), catalytic hairpin assembly (CHA), and hybridization chain reaction (HCR).^33–35^ Among these, TMSD triggers branch migration *via* single-stranded toehold binding to complete strand displacement. Compared to CHA and HCR, TMSD offers greater controllability of reaction kinetics and more flexible sequence design.^36,37^ Presently, the TMSD strategy has been employed in analysis of many intracellular targets, including miRNA.^38–40^

In this study, a novel HClO imaging strategy based on TMSD-mediated signal amplification was developed. This method utilizes a DNA duplex complex (TN) assembled by three single strands as the detection entity, incorporating a hairpin HP (containing a phosphorothioate site, PS site, which can be specifically cleaved by HClO) and a single strand T-B to specifically recognize HClO and drive the cyclic reaction, respectively (Scheme 1). When HClO participates in the reaction, HP is oxidized and cleaved, releasing the active sequence within it, which can then invade TN, initiating the TMSD reaction. Subsequently, the reaction cycle is sustained with the cooperation of T-B. This ultimately leads to the release of a short fragment with a FAM group at the terminal end in TN, resulting in enhanced fluorescence of the system. Thus, the fluorescence intensity can directly reflect the concentration of HClO. Notably, experimental results demonstrate the efficacy of the proposed method, exhibiting high sensitivity (LOD of 12.9 nM), high specificity (strong resistance to interference), and high reaction efficiency (the detection process takes only 20 minutes). The method also shows stable performance in the analysis of real samples. In addition, intracellular imaging experiments validate the effectiveness of this method, enabling the reliable differentiation between cancer cells and normal cells. These offer it great potential for application in the early diagnosis of cancer.

**Scheme 1:**
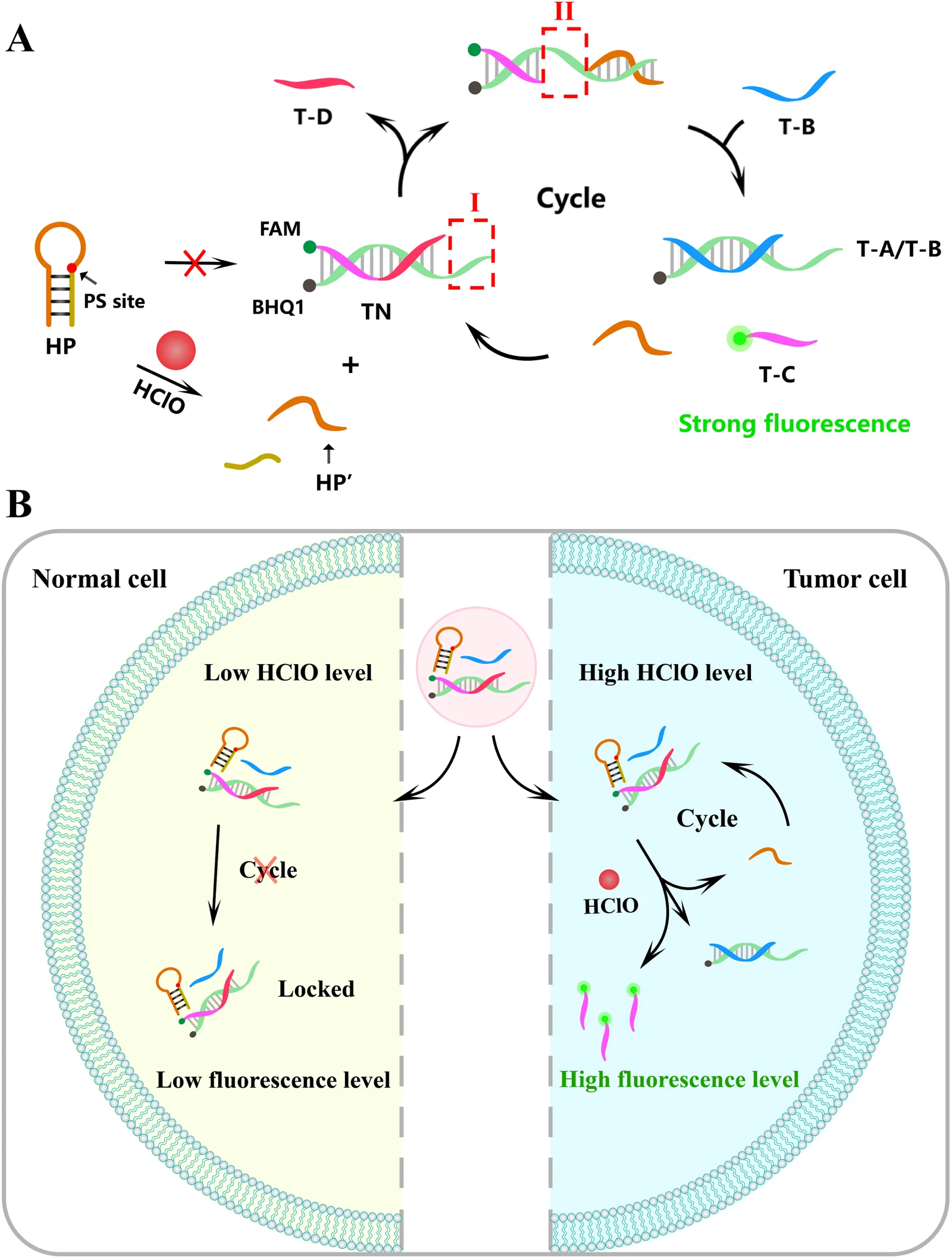
Working principle of the HClO detection strategy proposed. (A) The specific response mechanism of the strategy towards HClO. (B) The capability for distinguishing cancer cells from normal cells.

## Experimental

### Materials

DNA sequences utilized in the study were obtained from Sangon Biotechnology Co., Ltd (Shanghai, China), and were annotated in Table S1. Sodium hypochlorite (NaClO) was procured from Sigma-Aldrich Co. LLC (St. Louis, MO, USA, Cat. No. 239305, available chlorine was calibrated by iodometric titration before use). The materials used for gel preparation and the nucleic acid stain (NA-Red) were obtained from Biyuntian Biotechnology Co., Ltd (Shanghai, China). Chemical reagents such as sodium nitrate (NaNO_3_), hydrogen peroxide (H_2_O_2_), and magnesium chloride (MgCl_2_) were purchased from Sinopharm Chemical Reagent Co., Ltd (Shanghai, China). Human serum was obtained from PAN-Biotech Co., Ltd (Adenbach, Germany). Cells including SNU387, PC9 and LO2 were obtained from Zhong Qiao Xin Zhou Biotechnology Co., Ltd (Shanghai, China). The water used in experiments was ultrapure water (resistivity greater than 18.2 MΩ cm).

### Apparatus

Fluorescence intensity measurements were performed by F-2700 fluorescence spectrophotometer (Hitachi, Japan). The excitation and emission wavelengths were set to 495 nm and 510–610 nm, respectively, with slit widths of 5.0 nm and 10 nm. The fluorescence intensity at 525 nm was selected for analysis.

### TN assembly and HP preparation

TN assembly: the strands T-A, T-C, and T-D were mixed in equal proportions (1 : 1 : 1) in 50 mM Tris buffer (10 mM MgCl_2_, pH 7.4). The mixture was treated sequentially at 95 °C for 5 minutes, 4 °C for 10 minutes, and 37 °C for 1 hour to assemble the three-chain complex. The prepared TN was stored at 4 °C. HP preparation: HP was dissolved in 50 mM Tris buffer (10 mM MgCl_2_, pH 7.4), denatured at 95 °C for 5 minutes, and then slowly cool to room temperature to induce hairpin structure formation. The prepared HP was stored at 4 °C.

### Native polyacrylamide gel electrophoresis

A total 20 µL mixture of DNA samples and 6X loading buffer was utilized for electrophoresis. The mixture was loaded onto a 20% native polyacrylamide gel and electrophoresed at 150 V for 1.5 hours. Subsequently, the gel was stained with the nucleic acid dye NA-Red for 30 minutes.

### HClO detection

50 nM HP, 400 nM TN, 400 nM T-B and different concentrations of HClO were mixed in 50 mM Tris buffer (10 mM MgCl_2_, pH 7.4). The mixture was then incubated at 37 °C for 20 minutes. After that, the fluorescence intensity of each group was measured. The total volume of the reaction system was 50 µL.

### Cell culture and preparation of cell lysate

Cells were cultivated in RPMI-1640 medium (5% CO_2_, 37 °C). Following the harvesting of the cells with PBS, a method involving repeated freeze–thaw cycles with liquid nitrogen and a 25 °C water bath was employed to lyse the cells. Then, a centrifugal process at 12 000 rpm (4 °C, 30 minutes) was conducted. Finally, the upper layer was collected.

### Cell imaging

HP (200 nM), TN (600 nM), T-B (600 nM) and liposomes (SuperKine™ Lipo3.0 Transfection Reagent (Abbkine Scientific, Wuhan, China)) were mixed and then added to the cell culture medium, incubated with cells for 4 hours. Following a staining procedure with Hoechst 33 342 (10 µg mL^−1^), the cells were observed under a fluorescence microscope.

## Results and discussion

### Working principle of the HClO detection strategy

As shown in Scheme 1, this strategy utilizes a hairpin DNA (HP), a three-chain complex (TN), and a single-stranded DNA (T-B) to detect HClO. The TN is composed of three individual single-stranded DNA chains, designated as T-A, T-C, and T-D. The termini of T-A and T-C are labeled with the quencher BHQ1 and the fluorophore FAM, respectively. When the system exhibites no reaction, the fluorescence of FAM is quenched by BHQ1 due to their spatial proximity. In the presence of HClO, the PS site on HP will be oxidized and cleaved, releasing the active sequence HP’. HP’ can invade the toehold domain I at the terminus of TN, triggering a toehold-mediated strand displacement reaction and displacing T-D. Meanwhile, this will expose an additional toehold domain II. T-B can rapidly bind to domain II, forming a stable T-A/T-B double-strand complex and releasing HP’ and T-C, which results in a significant increase in system fluorescence. Concurrently, the released HP’ can proceed to invade other TN, thereby instigating a subsequent round of displacement reactions and attaining signal amplification. However, when HClO is absent, HP, TN, and T-B stably coexist. The cyclic reaction cannot be initiated, resulting in a weak fluorescence background. This difference in fluorescence enables quantitative analysis of HClO. Notably, this strategy combines HClO-PS site-specific recognition and the TMSD cycle, which can occur simultaneously within the system. The detection process involves only one reaction step. These can significantly improve detection efficiency and reduce system complexity.

### Feasibility analysis of HClO detection

To ascertain the practical viability of this strategy, four distinct reaction systems were established for analysis. As demonstrated in Fig. 1A, the fluorescence of the system exhibited a substantial increase exclusively when HP, TN, T-B, and HClO were present concurrently (curve *a*). All other groups showed minimal fluorescence levels. When HP, TN, and T-B were present simultaneously, the fluorescence level was found to be marginally elevated in comparison to the other two groups (curve *b*). This phenomenon is attributed to a small proportion of HP structure dissociating and binding to TN. Furthermore, native polyacrylamide gel electrophoresis was employed to characterize this method. As illustrated in Fig. 1B, lanes 2–7 exhibit the HP hairpin, the four individual strands T-A → T-D, and the corresponding TN band, respectively. A new, faint band appeared in lanes 8 and 9, situated beneath the TN band. This band corresponds to the T-A/T-B double-strand complex, which forms by a small proportion of T-B directly displacing T-C and T-D within the TN structure. Only in lane 10, TN was almost entirely converted into T-A/T-B double-strand complex, accompanied by the emergence of two new bands beneath the HP band and T-C band, respectively. These bands signify the two short chains resulting from the cleavage of HP by HClO. Overall, these results indicate the successful completion of the reaction and demonstrate the practical feasibility of this strategy.

**Fig. 1:**
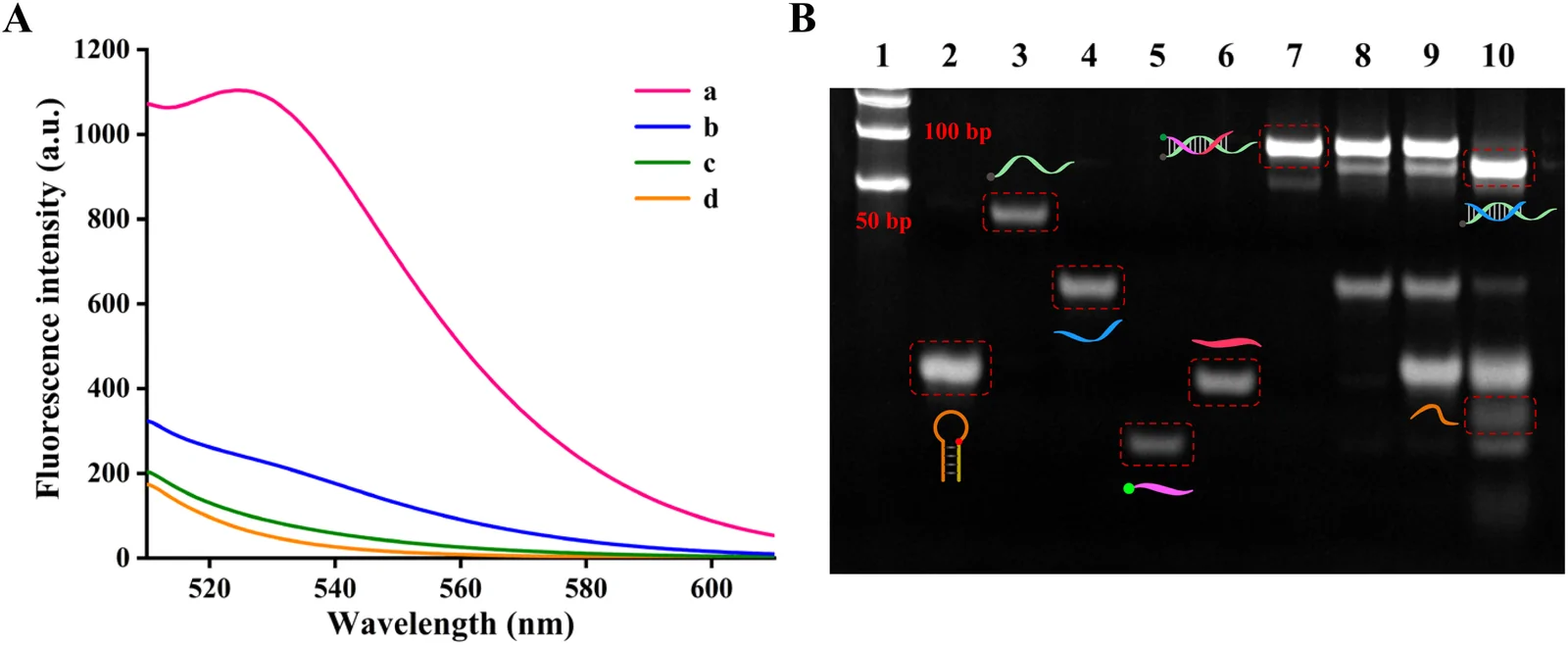
Feasibility study. (A) Fluorescence spectra of different systems. (a) TN + T-B + HP + HClO; (b) TN + T-B + HP; (c) TN + T-B; (d) TN. Concentrations of TN, T-B, HP were 600 nM while HClO was 50 µM. (B) Electrophoretic characterization. Lane 1, marker; lane 2, HP; lane 3, T-A; lane 4, T-B; lane 5, T-C; lane 6, T-D; lane 7, TN; lane 8, TN + T-B; lane 9, TN + T-B + HP; lane 10, TN + T-B + HP + HClO. The full uncropped gel image is provided in Fig. S9 of the SI.

### Evaluation of the detection performance for HClO

Firstly, to investigate the operating pH range of this sensing system, experiments were conducted using Tris buffers with pH values of 5.4, 6.4, 7.4, 8.4, and 9.4 to analyze changes in fluorescence intensity in the presence and absence of HClO. As demonstrated in Fig. S1, the probe demonstrates a satisfactory fluorescence response when the system pH was maintained within the range of 7.4 to 9.4. Notably, when the pH level gradually decreased from 7.4 to 5.4, the upper limit of the probe fluorescence response toward HClO experienced a significant decrease. At a pH of 5.4, HClO induced only a negligible fluorescence response from the probe. These results indicate that this detection probe can maintain satisfactory detection capability only within a specific pH range.

Then, before evaluating the detection performance of this method, the experimental conditions were optimized, including the concentrations of HP, TN, and T-B, and the total reaction time. The results showed that when the concentrations of HP, TN, and T-B were 50 nM, 400 nM, and 400 nM, respectively, and the reaction time was 20 minutes, the fluorescence intensity ratio between the systems with and without HClO (*F*/*F*_0_) was appropriate (Fig. S2–S5). Thus, these conditions were selected as the optimal parameters for subsequent experiments.

Afterthat, the optimal reaction system was employed for the quantitative analysis of HClO. HClO of varying concentrations (0, 0.01, 0.05, 0.1, 0.5, 1, 2, 3, 5, 7, 10, 20, 30, 50 µM) was utilized in the reaction, and the fluorescence intensities of each group were compared. As illustrated in Fig. 2A, the fluorescence spectra are presented. As the HClO concentration increased, the curves shifted upward, and the fluorescence intensity increased. A strong linear correlation was identified between the fluorescence intensity and the HClO concentration (*y* = 53.233*x* + 53.276, *R*^2^ = 0.995), within a range of concentrations from 0.01 to 7 µM (Fig. 2B). The limit of detection (LOD) was determined to be 12.9 nM (3*σ*/*S*, *n* = 9, which represents 9 independent replicate experiments performed on blank samples). Additionally, the specificity of the method for HClO was assessed. Interference experiments were conducted using other substances, including hydrogen peroxide (H_2_O_2_) and superoxide anion (O_2_^−^). The results demonstrated that only HClO triggered a substantial increase in fluorescence within the system, while fluorescence in other groups persisted at low levels (Fig. 3A). Furthermore, the addition of HClO to systems containing interfering substances resulted in an augmentation of fluorescence intensity, approaching the level observed in system containing HClO alone (Fig. 3B). These results confirm the excellent HClO specificity of the method.

**Fig. 2:**
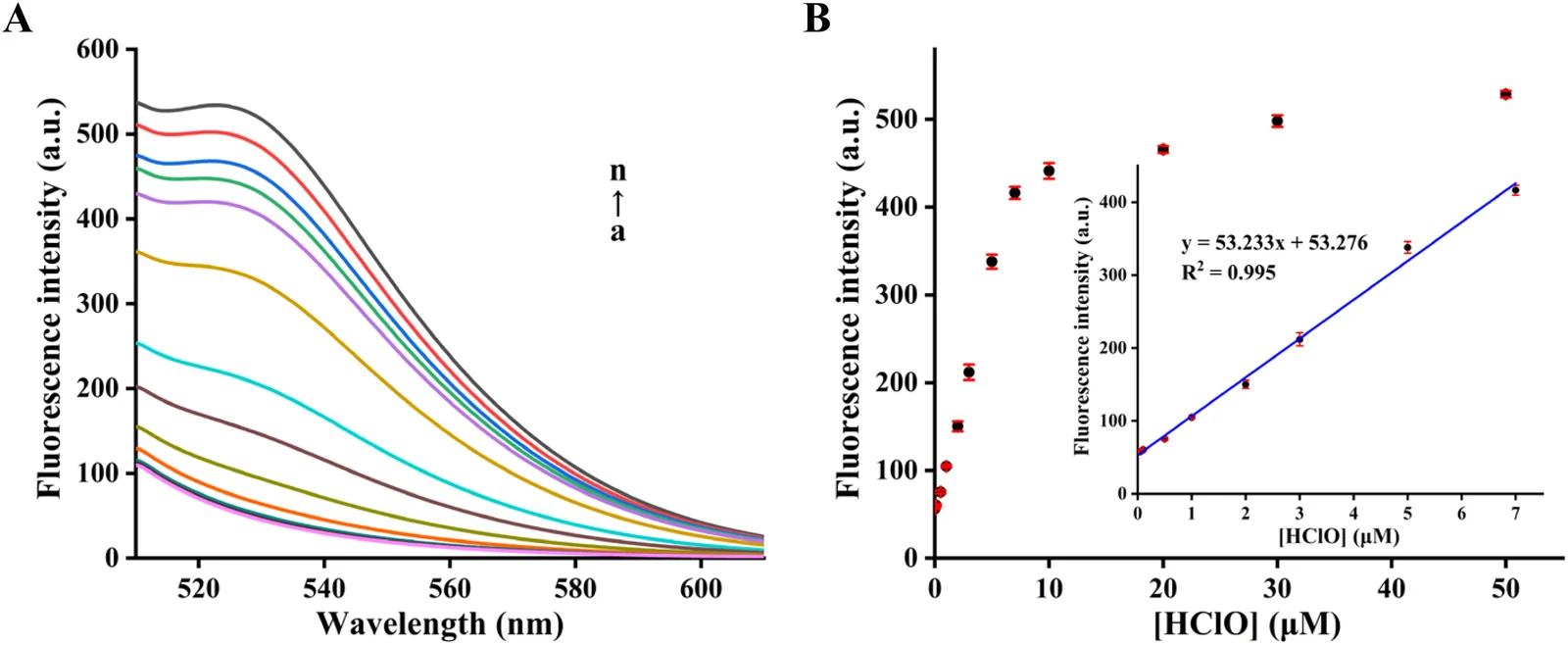
Quantitative analysis of HClO. (A) Fluorescence spectra for groups of different HClO concentrations. a → n: 0, 0.01, 0.05, 0.1, 0.5, 1, 2, 3, 5, 7, 10, 20, 30, 50 µM. (B) Fluorescence intensity values in (A). The inset shows the linear relationship between fluorescence intensity and HClO concentration. Data are presented as mean ± sd (*n* = 3).

**Fig. 3:**
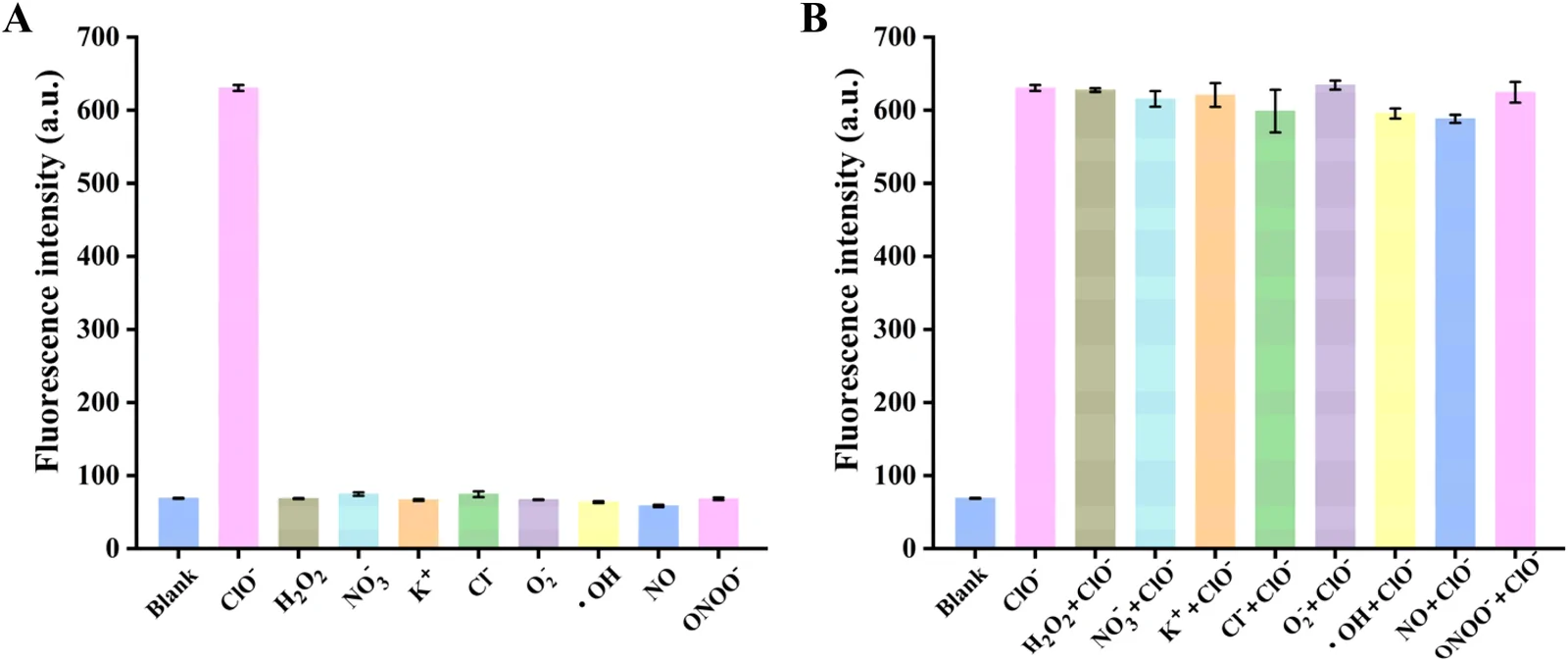
Specificity assessment. Comparison of fluorescence intensities between the system with interfering substances before (A) and after (B) the addition of HClO and the HClO system. Concentrations of all substances were 50 µM. Data are presented as mean ± sd (*n* = 3).

In summary, the detection performance of this method exceeded or is comparable to that of numerous existing methods (Table S2). Compared to probes based on silicon–rhodamine and coumarin–benzopyrylium hydrazide,^41,42^ this method significantly increases the sensitivity of detection. Additionally, the synthesis of the DNA strands used in this method is relatively simple, unlike the complex preparation processes involved in the gold nanoparticle-based detection method.^31^ The entire detection process consists of a single-step cyclic reaction, greatly reducing the complexity of the detection system and improving the detection efficiency while effectively avoiding the need for cumbersome manual intervention. These advantages lay a solid foundation for its subsequent practical application.

### Detection capability testing in real samples

Then, to validate the comprehensive detection performance of this method in real samples, recovery experiments were conducted in tap water, 2% diluted human serum, and SNU387 cell lysate. As demonstrated in Table S3, apparent concentrations of 337 nM and 798 nM of HClO were measured in tap water and cell lysate, respectively. This corresponds to the presence of residual disinfectant in the tap water and of endogenous HClO within the cells. And no HClO was found in serum. Notably, the HClO recovery rates ranged from 92.2% to 107.8% across all the three samples, confirming the analytical accuracy. This indicates the reliable performance of the method for real sample analysis, providing direct evidence for its application potential in clinical diagnosis.

### Intracellular HClO imaging for cancer cells and normal cells distinguishing

Finally, the method was employed to image and differentiate between HClO in cancer cells and normal cells. The experiments were conducted using three types of cell: SNU387 liver cancer cells, PC9 lung adenocarcinoma cells and LO2 normal liver cells. As demonstrated in Fig. 4A, the fluorescence signals in PC9 and SNU387 cells were notably more pronounced compared to that observed in LO2 cells, indicating elevated levels of HClO. The quantitative analysis results showed that there are differences in intracellular fluorescence levels between SNU387 and PC9 cells (Fig. 4B), which can be attributed to differences in their respective intracellular environments. To confirm that the increase in fluorescent signal in cancer cells was caused by HClO, the cells were pretreated with *N*-acetylcysteine (NAC, an ROS scavenger) to effectively eliminate intracellular HClO. As shown in Fig. S6 and S7, the intracellular fluorescence signals in PC9 and SNU387 cells were significantly reduced after NAC treatment. Given that the probe itself exhibits excellent specificity for HClO, these results collectively support that the enhancement of the fluorescence signals is indeed HClO-dependent. Moreover, the cytotoxicity of the probe used in this method was evaluated by the CCK-8 assay. Fig. S8 shows that co-incubation with the probe did not significantly affect cell viability, demonstrating its excellent biocompatibility. Overall, this method enables the imaging of intracellular HClO and can be used to distinguish cancer cells from normal cells, offering a promising new tool for the early diagnosis of cancer.

**Fig. 4:**
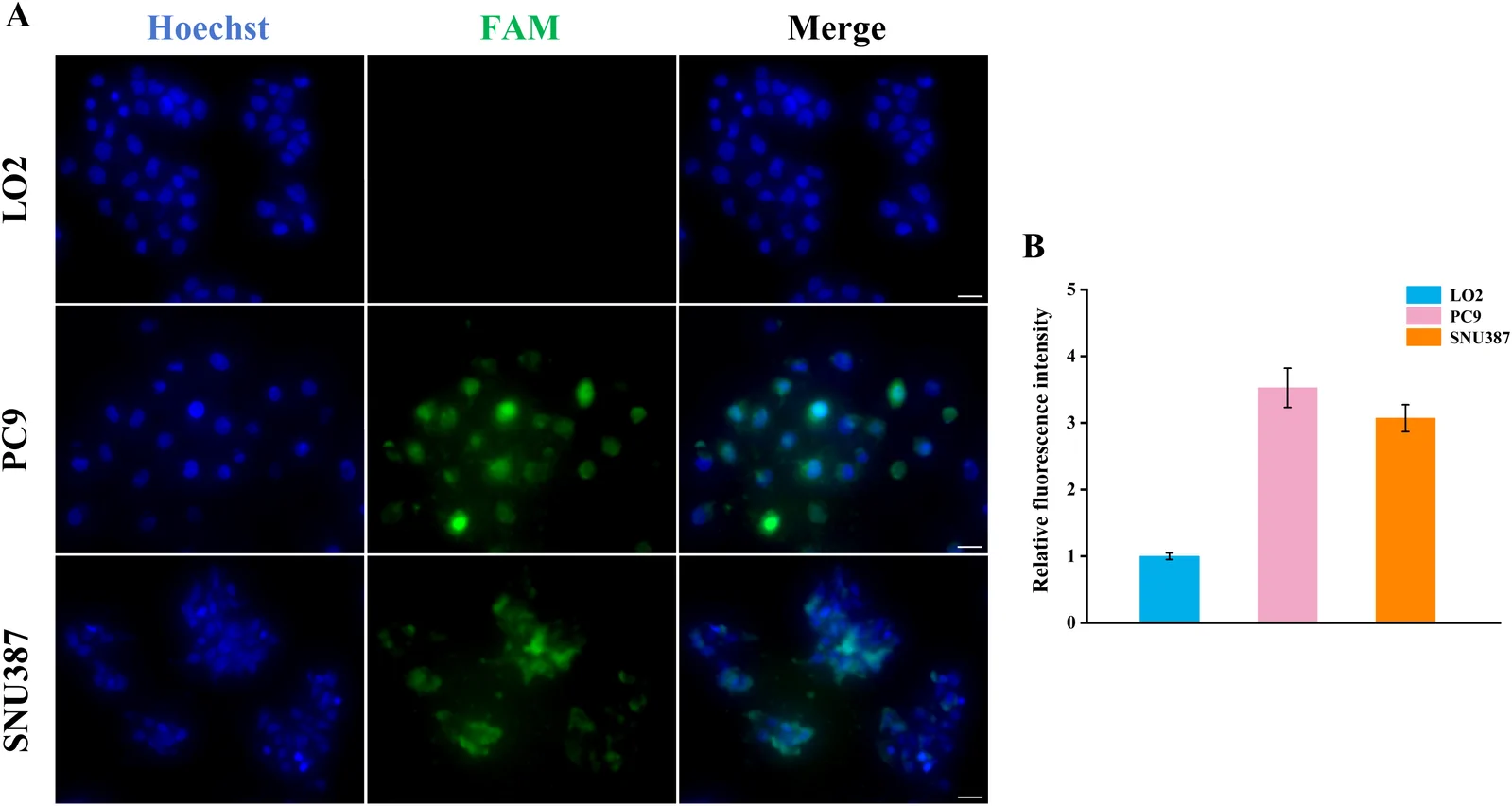
Intracellular HClO imaging. (A) Images of cells under a fluorescence microscope. (B) Relative fluorescence intensity in (A). Scale bar: 20 µm. Data are presented as mean ± sd (*n* = 3).

## Conclusions

This study developed an HClO-responsive fluorescence sensing strategy, which integrates PS-modified DNA recognition with TMSD-mediated signal amplification, for distinguishing cancer cells from normal cells. The detection probe in this method consists of the hairpin HP, the three-chain complex TN, and the single-stranded T-B. In the presence of HClO, HP is specifically cleaved, releasing the active sequence HP’. HP’ initiates the cyclic reaction by invading the TN terminus, while T-B plays a pivotal role in amplifying the signal by facilitating the cycling process. The method possesses benefits including high sensitivity (LOD of 12.9 nM), high specificity, high efficiency (the detection reaction takes only 20 minutes), simplicity (one-step reaction), and low cost. Recovery tests confirm its reliability and accuracy in real samples. Most importantly, this assay enables the distinction between cancer cells and normal cells. Given its excellent performance, this method holds great promise for applications in early cancer diagnosis. However, this method is suitable for end-point detection after a specific reaction time, with the end-point fluorescence signal reflecting the HClO level. It should be noted that this method has limitations when it comes to continuous, dynamic monitoring of changes in HClO levels. Meanwhile, this study involves a limited number of actual sample types and tumor cell types; further evaluation of more other samples is needed to verify its broader applicability. Additionally, due to the excellent programmability of DNA strands, this detection strategy can be used to develop detection methods for other targets, providing new insights in the field of biochemical analysis. Overall, this work proposed an effective and reliable method for HClO analysis, which enables distinguishing between cancer cells and normal cells, holding great application potential in early cancer diagnosis and biochemical analysis.

## Author contributions

Qianqian Duan: experiments, data curation, methodology, writing – original draft. Aihua Xu: experiments, data curation, methodology, writing – original draft. Ruobing Han: data curation, methodology. Guochao Xu: formal analysis. Qianqing Wang: conceptualization, supervision, writing – review & editing. Changbei Ma: supervision, project administration, writing – review & editing.

## Conflicts of interest

The authors declare that they have no known competing financial interests or personal relationships that could have appeared to influence the work reported in this paper.

## Supplementary Material

RA-OLF-D6RA08265C-s001

## Data Availability

The data that support the findings of this study are available from the corresponding author upon reasonable request. Supplementary information (SI) is available. See DOI: https://doi.org/10.1039/d6ra08265c.

## References

[cit1] Li W., Liang H., Wang W., Liu J., Liu X., Lao S., Liang W., He J. (2024). J. Hematol. Oncol..

[cit2] Mao G.-J., Zhang S.-Y., Yang T.-T., Zhu B., Sun X.-Y., Wang Q.-Q., Zhang G. (2024). Talanta.

[cit3] Finn R. S., Gu K., Chen X., Merle P., Lee K.-H., Bouattour M., Cao P., Wang W., Cheng A.-L., Zhu L., Lim H. Y., Kudo M., Pan Y., Chang T.-T., Edeline J., Li W., Yang P., Li C., Li J., Siegel A. B., Qin S. (2025). JHEP Rep..

[cit4] Zarinshenas R., Amini A., Mambetsariev I., Abuali T., Fricke J., Ladbury C., Salgia R. (2023). Cancers.

[cit5] Fitzgerald R. C., Antoniou A. C., Fruk L., Rosenfeld N. (2022). Nat. Med..

[cit6] Wang Y., Deng B. (2023). Cancer Metastasis Rev..

[cit7] McCormack V., Aggarwal A. (2021). Br. J. Cancer.

[cit8] Barzaman K., Karami J., Zarei Z., Hosseinzadeh A., Kazemi M. H., Moradi-Kalbolandi S., Safari E., Farahmand L. (2020). Int. Immunopharmacol..

[cit9] Valadez-Cosmes P., Raftopoulou S., Mihalic Z. N., Marsche G., Kargl J. (2022). Pharmacol. Ther..

[cit10] Wang W., Xiong J., Song X., Wang Z., Zhang F., Mao Z. (2020). Anal. Chem..

[cit11] Liang Y., Xu T., Xu S., Xu X., Wang L., Wang Z., Wang S. (2025). Biosens. Bioelectron..

[cit12] Chen P., Lu Y., He B., Xie T., Yan C., Liu T., Wu S., Yeh Y., Li Z., Huang W., Zhang X. (2023). Cell Death Dis..

[cit13] Cheung E. C., Vousden K. H. (2022). Nat. Rev. Cancer.

[cit14] Wang X., Wang R., Ding Q., Wu W., Che F., Li P., Zhang W., Zhang W., Liu Z., Tang B. (2022). Anal. Chem..

[cit15] Cui J., Teng J., Xiang P., Liu F., Cao Z., Lu J., Deng Y., Li Y., Peng C., Dehaen W., Fang Y. (2025). J. Hazard. Mater..

[cit16] Sun M., Wang Y., Xu H., Shen Y., Liu B., Ma Y., Jiang C., Wang S., Li Q., Lu Y., Han F., Li T., Qin Y. (2025). J. Med. Chem..

[cit17] Zheng H., Peng W., Liu M., Zhang S., Song X., Yang L. (2024). Anal. Chem..

[cit18] Yao L., Song H., Yin C., Huo F. (2024). Chem. Commun..

[cit19] Ye Z., Ji M., Wu K., Yang J., Liu A. A., Sun W., Ding D., Liu D. (2022). Angew. Chem., Int. Ed..

[cit20] Gui L., Yan J., Zhao J., Wang S., Ji Y., Liu J., Wu J., Yuan K., Liu H., Deng D., Yuan Z. (2023). Biosens. Bioelectron..

[cit21] Yuan F., Zhang S., Wang Y., Gao X., Zhao Y., Ning L., Wang Y., Guo Y., Zhang J. (2024). Anal. Chem..

[cit22] Zhu J., Miao C., Wang X. (2023). Spectrochim. Acta, Part A.

[cit23] Xu Q., Jiao C., Liu X., Yang Y., Ren J., Wang P., Pan X., Wu Y., Yan Z., Zeng J. (2025). J. Hazard. Mater..

[cit24] Yan L., Yang H., Li J., Zhou C., Li L., Wu X., Lei C. (2022). Anal. Chim. Acta.

[cit25] Fang X., Jin X., Ma X., Guan L., Chen W., She M. (2022). Anal. Bioanal. Chem..

[cit26] Thiruppathi M., Velusamy N., Lin P.-Y., Ho J.-a. A. (2026). Biosens. Bioelectron..

[cit27] Wang Y., Li W., Zhou P. (2024). Spectrochim. Acta, Part A.

[cit28] Fang H., Chen Y., Geng S., Yao S., Guo Z., He W. (2022). Anal. Chem..

[cit29] Long Y., Liu J., Ju Z., Qi F., Tang W., Yan S., Dai F., Zhang S., Zhou B. (2024). Anal. Chem..

[cit30] Ma J., Kong X., Zhao M., Jiao Z., Xie H., Si W., Li H., Zhang Z. (2024). Anal. Chim. Acta.

[cit31] Wu K., Yao C., Yang D., Liu D. (2022). Biosens. Bioelectron..

[cit32] Zhao J., Gao J., Xue W., Di Z., Xing H., Lu Y., Li L. (2018). J. Am. Chem. Soc..

[cit33] Song Y., Mao C., Zhang W., Deng D., Chen H., Sun P., Liu M., Feng C., Luo L. (2024). Biosens. Bioelectron..

[cit34] Li L., Xu S., Chen M., Lu P., Wang Y., Liu C., Jiao J., Miao P., Jiao J. (2025). Nano Today.

[cit35] Bai S., Xu B., Wu J., Xie G. (2023). Biosens. Bioelectron..

[cit36] Tang L., Luo T., Fan S., Liu Y., Song J. (2023). Biomater. Sci..

[cit37] Mayer T., Oesinghaus L., Simmel F. C. (2022). J. Am. Chem. Soc..

[cit38] Wang Q., Yu L., Peng Y., Sheng M., Jin Z., Zhang T., Huang J., Yang X. (2024). Anal. Chem..

[cit39] Marpaung D. S. S., Yap Sinaga A. O. (2026). Talanta.

[cit40] Li S., Zhu L., Lin S., Xu W. (2023). Biosens. Bioelectron..

[cit41] Liang F., Jiang J., Yang X., Zhang G., Zhou J., Han J., Geng Y., Wang Z. (2023). Chem. Commun..

[cit42] Huang W., Du X., Zhang C., Zhang S., Zhang J., Yang X.-F. (2022). Anal. Chem..

