# Supplementary material for: An efficient hypochlorous acid fluorescence imaging strategy based on toehold-mediated strand displacement for distinguishing cancer cells from normal cells
Source: RSC Adv. 2026 Oct 30. Online ahead of print. doi: 10.1039/d6ra08265c (PMC13720905; doi:10.1039/d6ra08265c)
Supplement: Supplementary file 1 [file RA-OLF-D6RA08265C-s001.pdf]

Supplementary Information (SI) for RSC Advances.
This journal is © The Royal Society of Chemistry

Supplementary Information

An efficient hypochlorous acid fluorescence imaging strategy based on toehold-mediated strand displacement for distinguishing cancer cells from normal cells

Qianqian Duan [1, #], Aihua Xu [1, 2, #], Ruobing Han [3], Guochao Xu [1], Qianqing Wang [3, 4, *], Changbei Ma [2, *]

[1] Department of Clinical Laboratory, Xinxiang Central Hospital, 453000, Xinxiang, Henan, China

[2] School of Life Sciences, Central South University, 410013, Changsha, Hunan, China

[3] Central Laboratory, Xinxiang Central Hospital, 453000, Xinxiang, Henan, China

[4] Department of Gynecology Oncology, Xinxiang Central Hospital, 453000, Xinxiang, Henan, China

[#] These authors contributed equally to this work.

[*] Corresponding author: Wangq1111@126.com (Q. Wang); macb2012@csu.edu.cn (C. Ma).

Table S1 DNA Sequences used in the study.

Name	Sequence (5' to 3')
HP	CAA GTA TGC CAA AGA CAC TCG C*TTG GCA TAC TTG
T-A	GTG CGA GTG TCT TTG GCA TAC TTG TCA AGG CCG TAA GTT GTG TTT -BHQ1
T-B	AAA CAC AAC TTA CGG CCT TGA CAA GTA TGC CAA AGA
T-C	FAM- AAA CAC AAC TTA CGG
T-D	CCT TGA CCT TGA CAA GTA TGC CAA AGA C

'*' represents PS site.

Table S2 Comparison of fluorescence methods for HClO detection

Materials	Detection Range (μM)	LOD (nM)	Cellular applications	Reference
Silicon-rhodamine-based formohydrazide	0 - 60	230	RAW 264.7, HeLa; endogenous/exogenous HClO imaging	1
Coumarin-benzopyrylium hydrazide	0 - 16	35	HeLa, RAW 264.7; dual-channel endogenous/exogenous imaging	2
Gold nanoparticle and functional DNA	0.05 - 1	8.51	RAW 264.7; confocal & flow cytometry	3
Cationic rhodamine derivative and 2-methoxyphenothiazine	0 - 20	15.4	HepG2; mitochondrial HClO imaging	4
Benzocoumarin-based water-soluble probe	0 - 10	2.45	RAW 264.7; endogenous/exogenous HClO imaging	5
Analogs of Changsha dyes	0 - 10	28.3	RAW 264.7; endogenous/exogenous HClO imaging	6

PS-modified DNA and TMSD cycle	0.01 - 7	12.9	Distinguish cancer cells from normal cells	This work

Table S3 Recovery test in real samples

Samples	Added (μM)	Found (μM)	Recovery (%)	RSD (%)
Tap water	0	0.337 ± 0.002	\	0.726
	1	1.329 ± 0.043	99.1	3.216
	3	3.102 ± 0.066	92.2	2.129
	5	5.668 ± 0.221	106.6	3.893
Serum	0	Not found	\	\
	1	0.925 ± 0.026	92.5	2.784
	3	3.086 ± 0.082	102.9	2.645
	5	5.345 ± 0.270	106.9	5.060
Cell lysate	0	0.798 ± 0.019	\	2.361
	1	1.814 ± 0.025	101.6	1.385
	3	3.716 ± 0.041	97.3	1.110
	5	6.190 ± 0.269	107.8	4.353

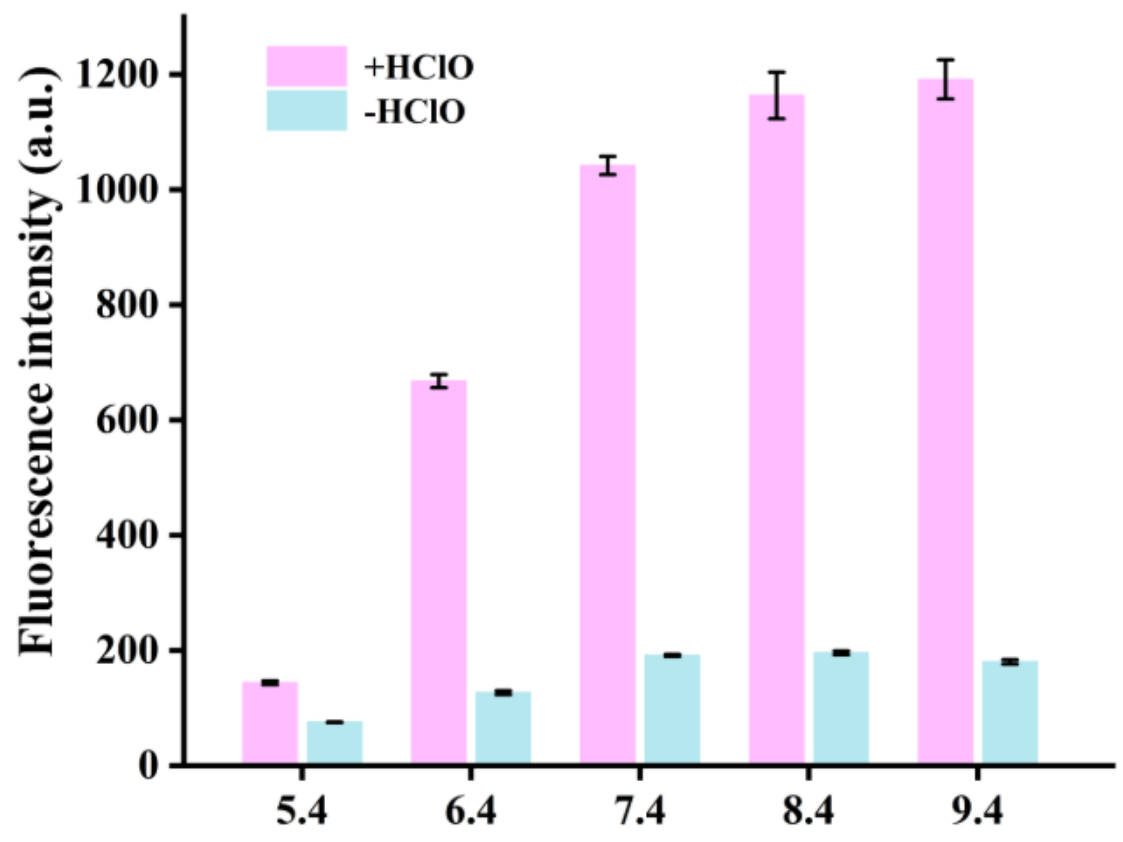

Fig. S1 Fluorescence response of the probe toward HClO under different pH conditions.

Data are presented as mean ± sd (n=3).

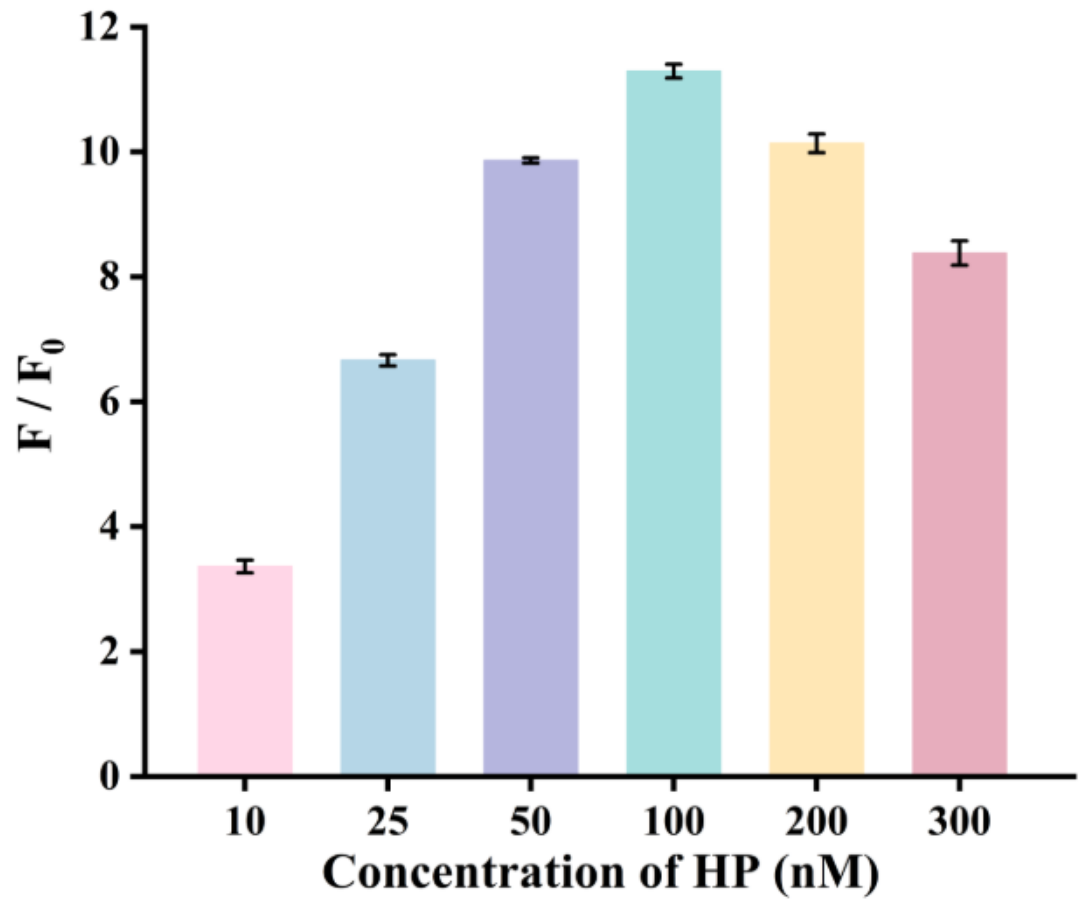

Fig. S2 Optimization of concentration of HP. F/F_0: The fluorescence intensity ratio between the systems with and without HClO. Data are presented as mean ± sd (n=3).

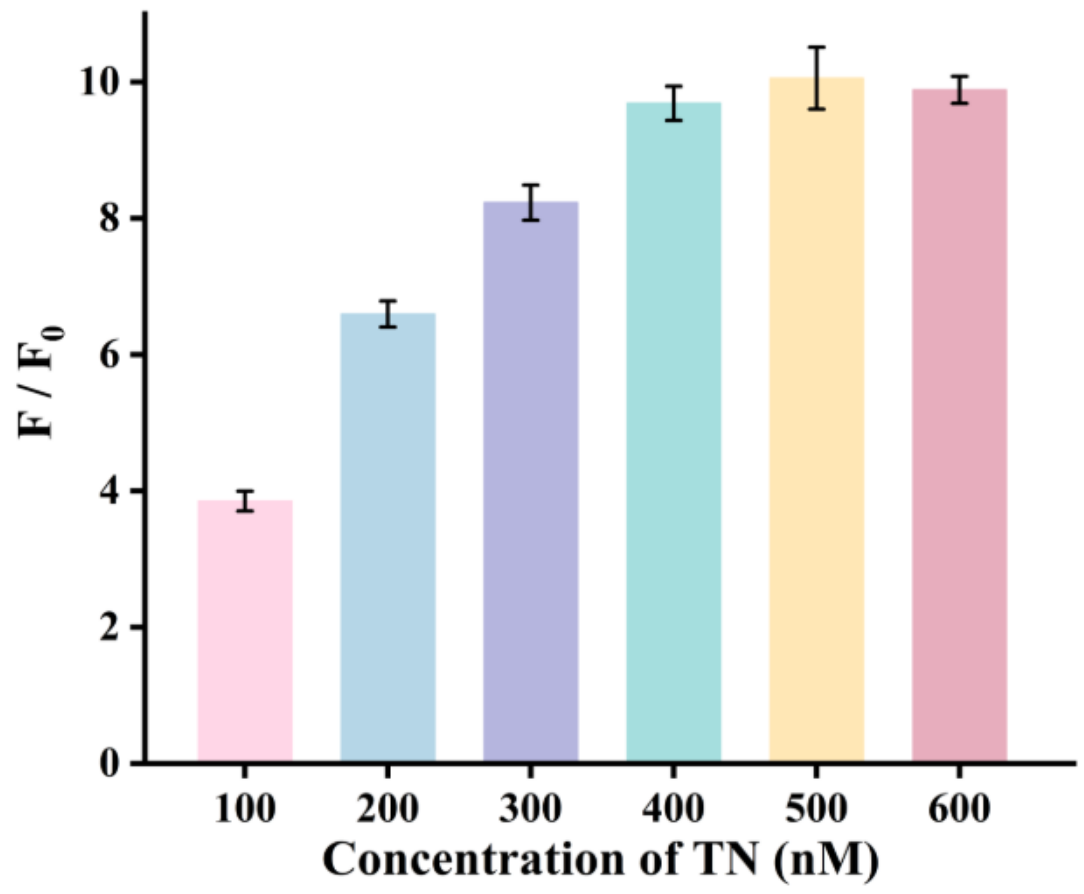

Fig. S3 Optimization of concentration of TN. F/F_0: The fluorescence intensity ratio between the systems with and without HClO. Data are presented as mean ± sd (n=3).

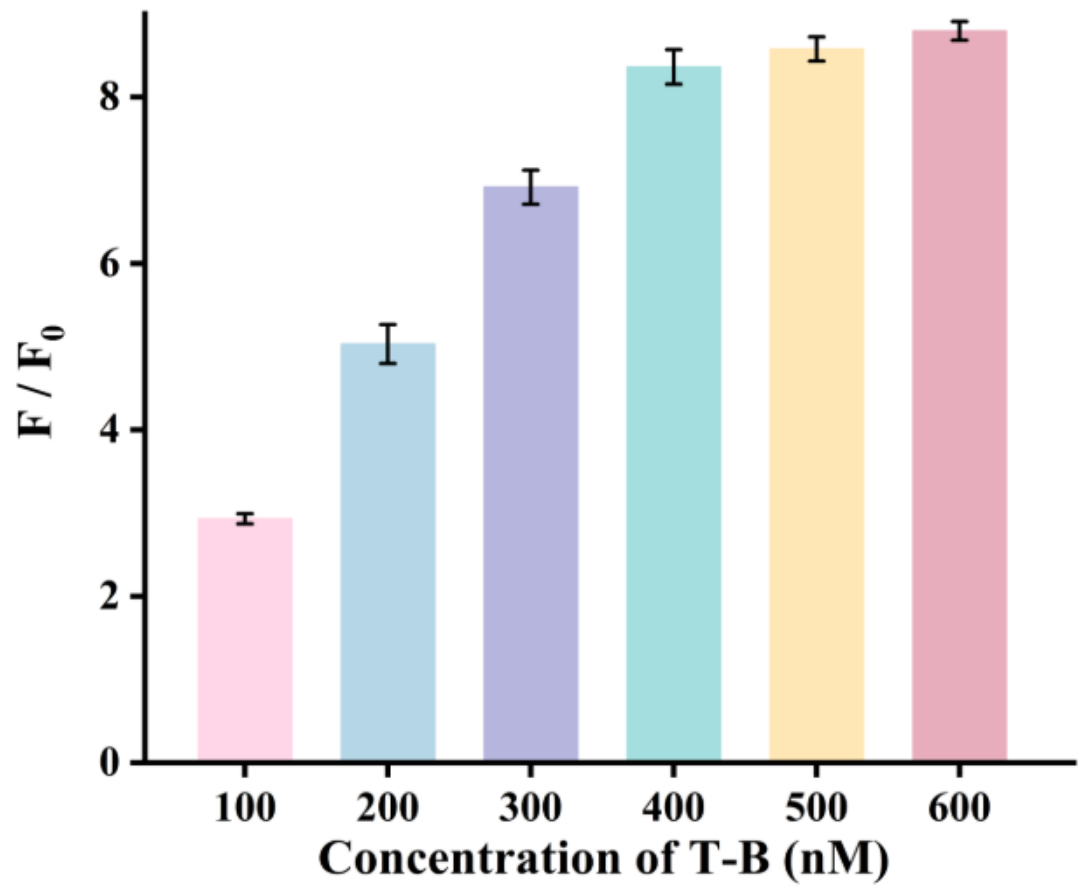

Fig. S4 Optimization of concentration of T-B. F/F_0: The fluorescence intensity ratio between the systems with and without HClO. Data are presented as mean ± sd (n=3).

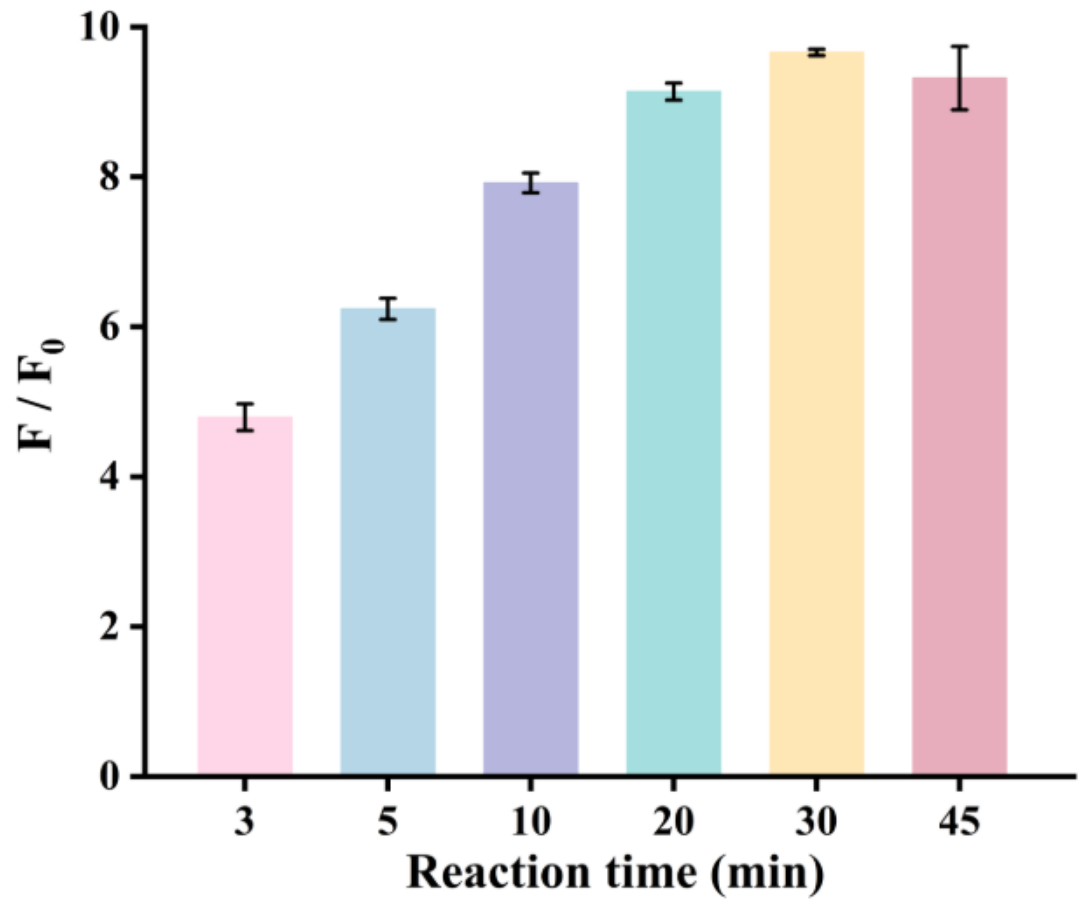

Fig. S5 Optimization of total reaction time. F/F_0: The fluorescence intensity ratio between the systems with and without HClO. Data are presented as mean ± sd (n=3).

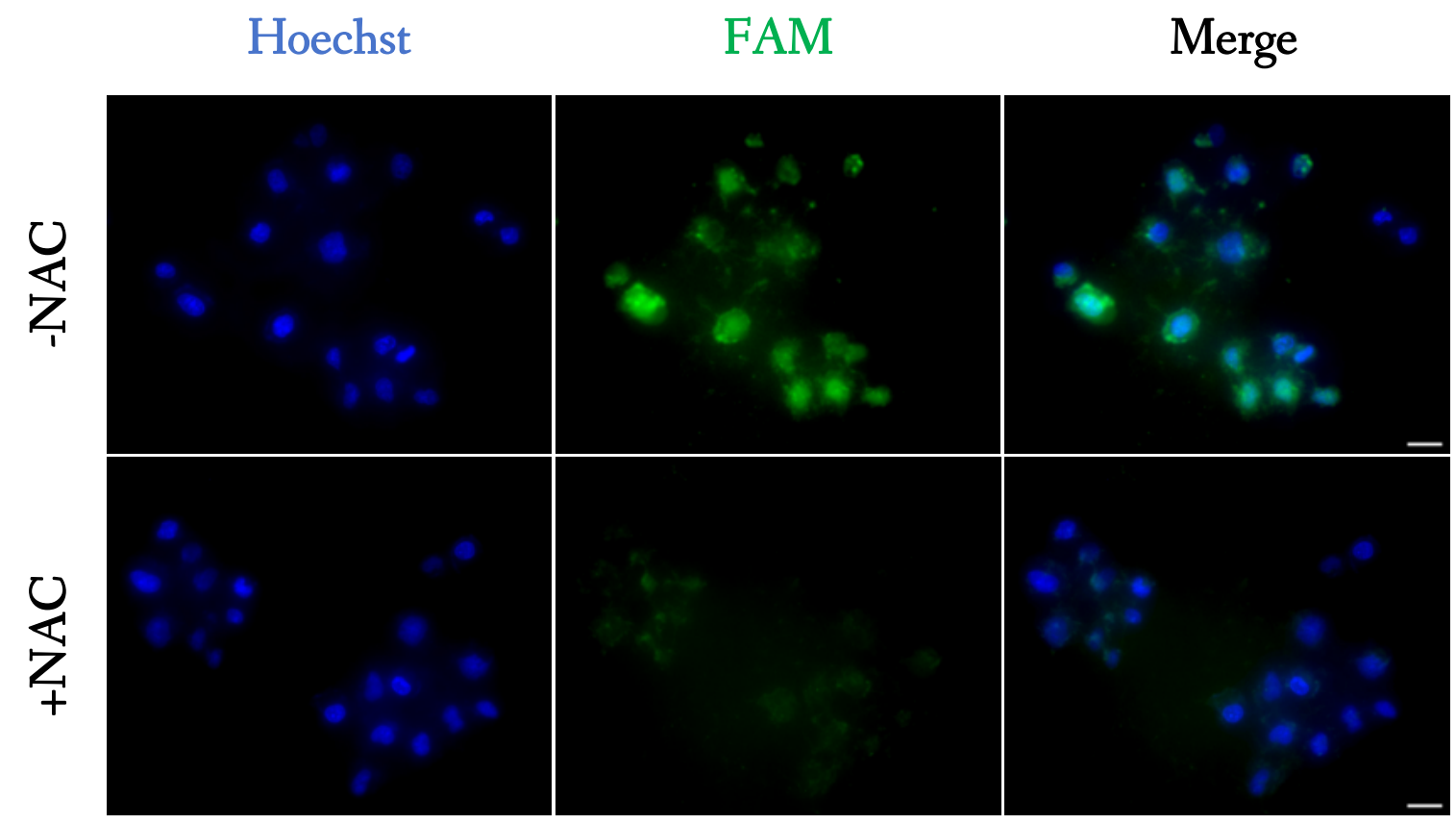

Fig. S6 Fluorescence images of PC9 cells with or without pretreatment of NAC. Scale bar: 20 μm.

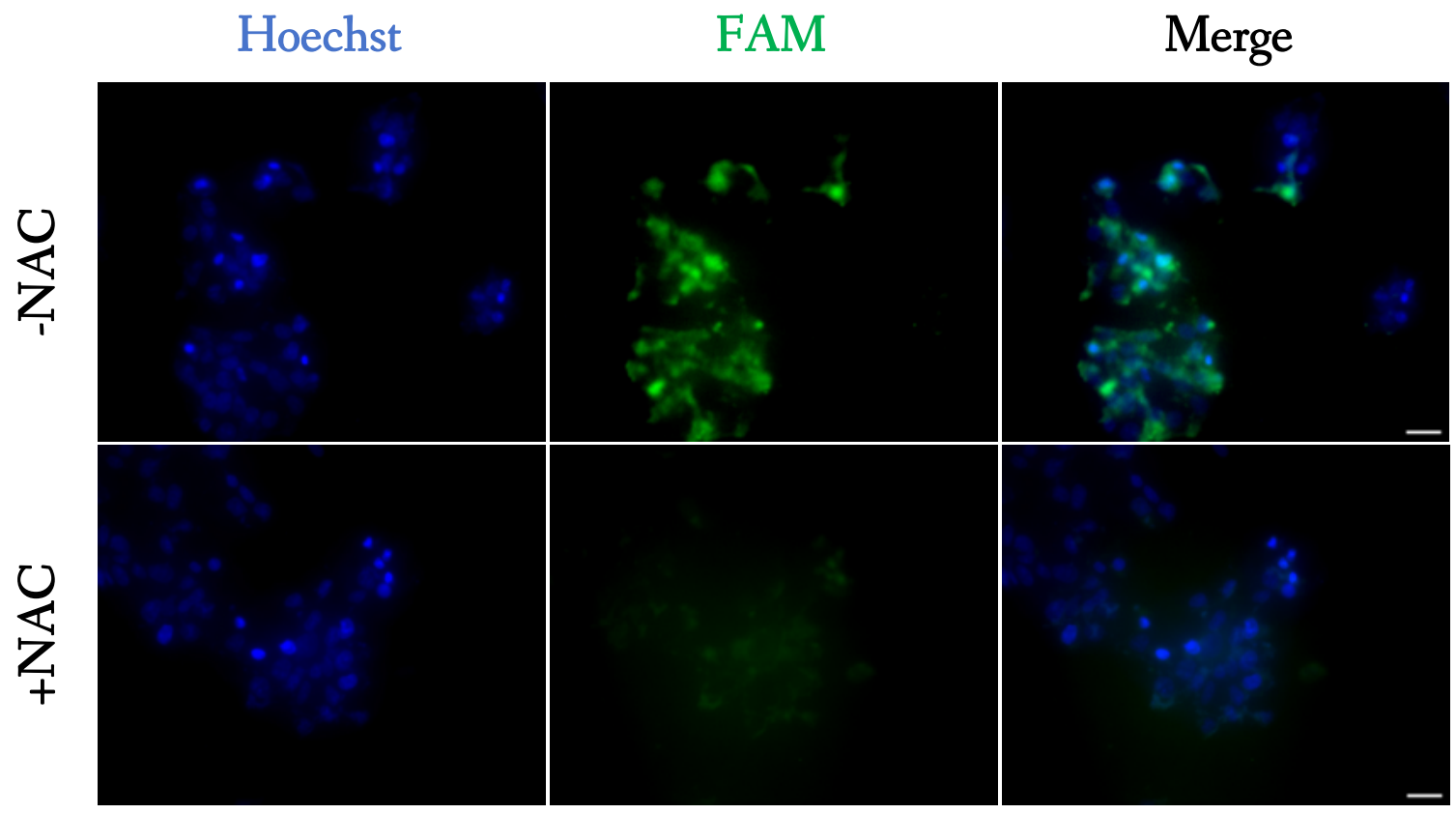

Fig. S7 Fluorescence images of SNU387 cells with or without pretreatment of NAC. Scale bar: 20 μm.

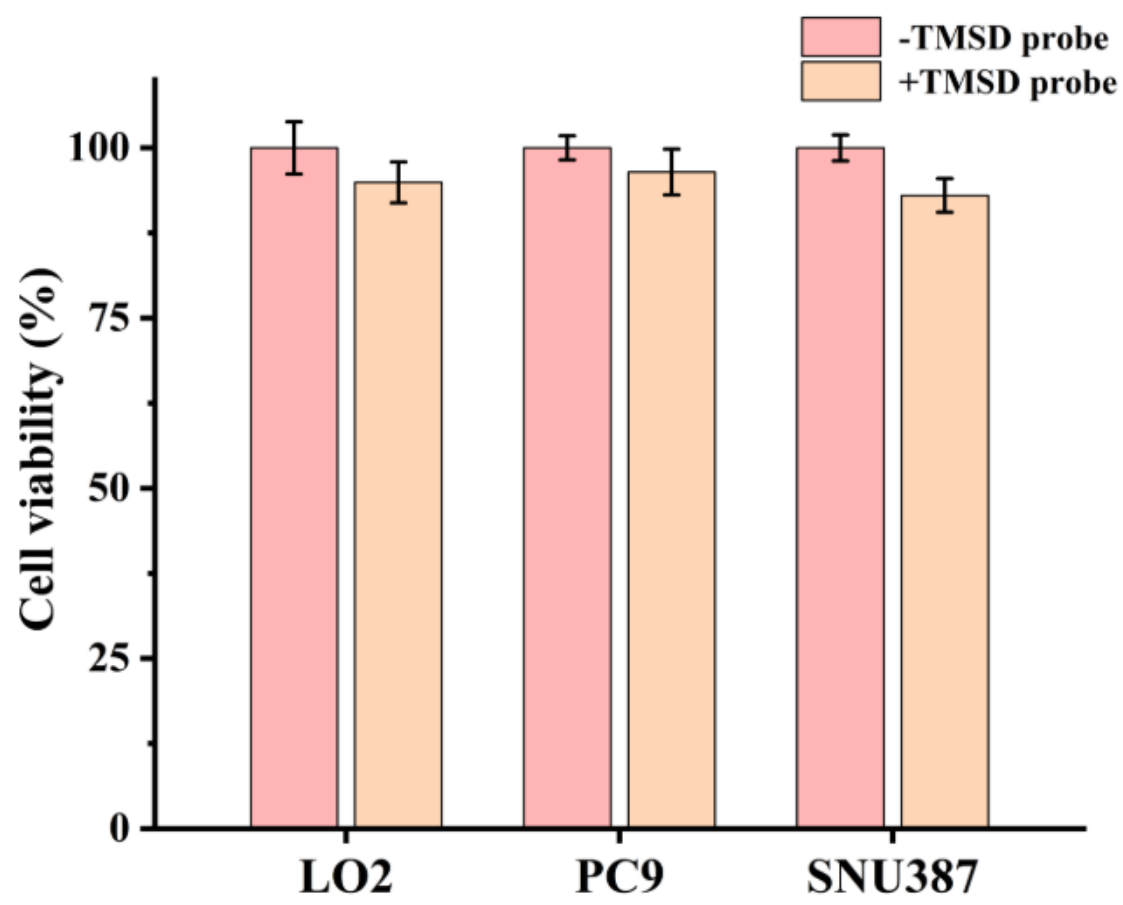

Fig. S8 Assessment of cell viability under different conditions using CCK-8 assay. Data are presented as mean ± sd (n=3).

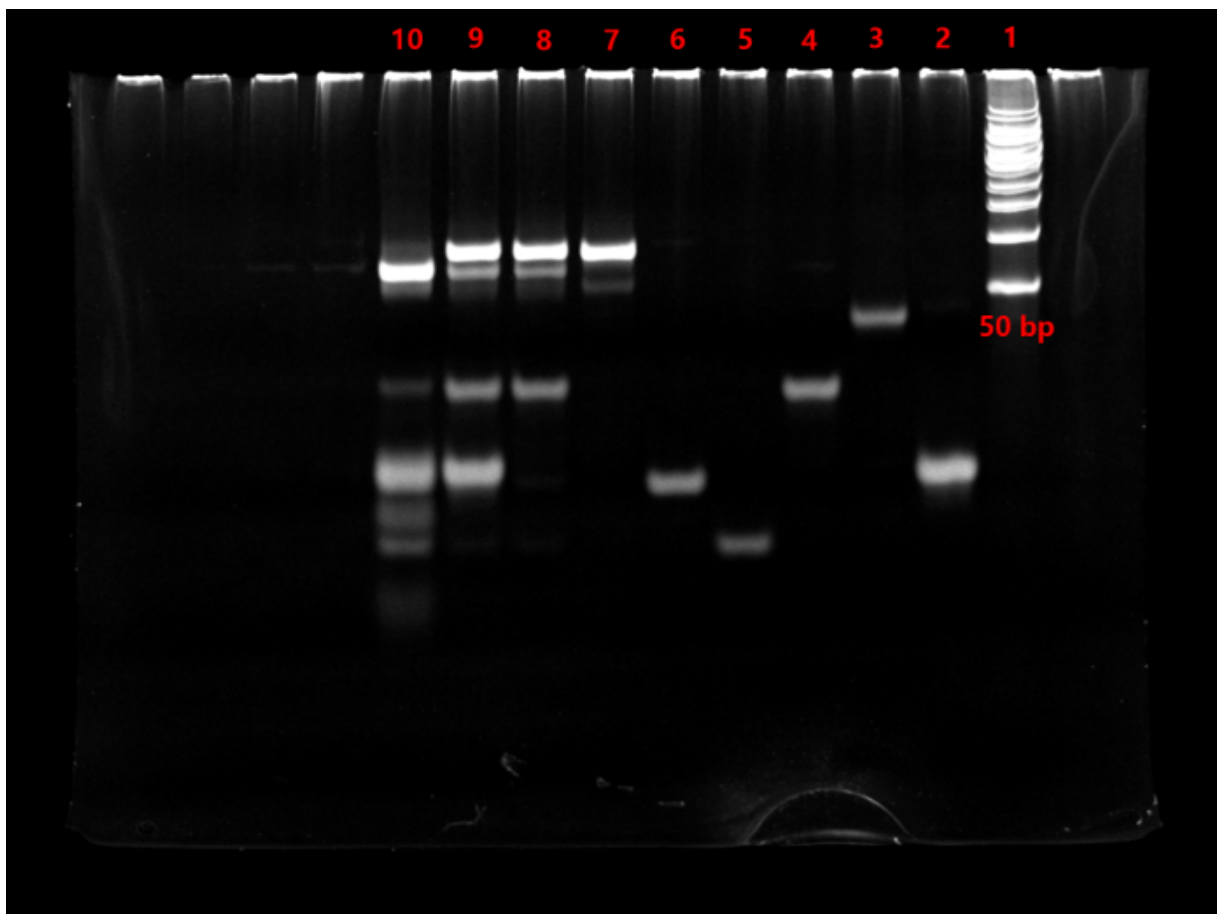

Fig. S9 Full uncropped raw image of gel corresponding to Fig. 1B. Lane 1, Marker; lane 2, HP; lane 3, T-A; lane 4, T-B; lane 5, T-C; lane 6, T-D; lane 7, TN; lane 8, TN+T-B; lane 9, TN+T-B+HP; lane 10, TN+T-B+HP+HClO.

References

1 F. Liang, J. Jiang, X. Yang, G. Zhang, J. Zhou, J. Han, Y. Geng and Z. Wang, *Chem. Commun.*, 2023, **59**, 1357-1360.

2 W. Huang, X. Du, C. Zhang, S. Zhang, J. Zhang and X.-F. Yang, *Anal. Chem.*, 2022, **94**, 17485-17493.

3 K. Wu, C. Yao, D. Yang and D. Liu, *Biosens. Bioelectron.*, 2022, **209**, 114273.

4 B. Zhao, X. Xu, X. Wen, Q. Liu, C. Dong, Q. Yang, C. Fan, J. Yoon and Z. Lu, *Anal. Chem.*, 2024, **96**, 5992-6000.

5 K. Liu, L. Fan, S. Huang, J. Sun, X. Wang, H. Li, C. Si, W. Zhang, T. Li and Z. Yang, *Analyst*, 2022, **147**, 1976-1985.

6 Q. Luo, Z. Luo, H. Zeng, Y. Xiao, Y. Peng and G. Liu, *Spectrochim. Acta A Mol. Biomol. Spectrosc.*, 2022, **273**, 121017.
